# Comparison between Conventional IMRT Planning and a Novel Real-Time Adaptive Planning Strategy in Hypofractionated Regimes for Prostate Cancer: A Proof-of-Concept Planning Study

**DOI:** 10.3390/healthcare7040153

**Published:** 2019-12-02

**Authors:** Maria Antico, Peter Prinsen, Alice Fracassi, Alfonso Isola, David Cobben, Davide Fontanarosa

**Affiliations:** 1 Philips Research, 5656 AE Eindhoven, The Netherlands; maria.antico@hdr.qut.edu.au (M.A.); Peter.Prinsen@philips.com (P.P.); ali.fracassi@gmail.com (A.F.); alfonso.isola@philips.com (A.I.); 2 Delft University of Technology, 2628 CD Delft, The Netherlands; 3 Institute of Health & Biomedical Innovation, Queensland University of Technology, Brisbane, QLD 4000, Australia; 4 School of Electrical Engineering and Computer Science, Queensland University of Technology, Gardens Point Campus, 2 George St, Brisbane, QLD 4000, Australia; 5 University of Rome Tor Vergata, 00133 Rome, Italy; 6 Division of Cancer Sciences, School of Medical Sciences, Faculty of Biology, Medicine and Health, University of Manchester, Manchester M13 9PL, UK; David.Cobben@christie.nhs.uk; 7 Department of Radiotherapy Related Research, University of Manchester, Manchester M13 9PL, UK; 8 The Christie National Health Trust, Wilmslow Road, Manchester M20 4BX, UK; 9 School of Clinical Sciences, Queensland University of Technology, Gardens Point Campus, 2 George St, Brisbane, QLD 4000, Australia

**Keywords:** adaptive rt, prostate cancer, radiotherapy, margin, intra-fraction motion, hypofractionation

## Abstract

In prostate cancer external beam radiation therapy (EBRT), intra-fraction prostate drifts may compromise the treatment efficacy by underdosing the target and/or overdosing the organs at risk. In this study, a recently developed real-time adaptive planning strategy for intensity-modulated radiation therapy (IMRT) for prostate cancer was evaluated in hypofractionated regimes against traditional treatment planning based on a treatment volume margin expansion. The proposed workflow makes use of a “library of plans” corresponding to possible intra-fraction prostate positions. During delivery, at each beam end, the plan prepared for the position of the prostate closest to the current one is selected and the corresponding beam delivered. This adaptive planning strategy was compared with the traditional approach on a clinical prostate cancer case where different prostate shift magnitudes were considered. Five, six and fifteen fraction hypofractionated schemes were considered for each of these scenarios. When shifts larger than the treatment margin were present, using the traditional approach the seminal vesicles were underdosed by 3–4% of the prescribed dose. The adaptive approach instead allowed for correct target dose coverage and lowered the dose on the rectum for each dosimetric endpoint on average by 3–4% in all the fractionation schemes. Standard intensity-modulated radiation therapy planning did not always guarantee a correct dose distribution on the seminal vesicles and the rectum. The adaptive planning strategy proposed resulted insensitive to the intra-fraction prostate drifts, produced a dose distribution in agreement with the dosimetric requirements in every case analysed and significantly lowered the dose on the rectum.

## 1. Introduction

In modern external beam radiation therapy (EBRT) for prostate cancer, intensity-modulated radiation therapy (IMRT) is currently the most common treatment technique, allowing for delivery of highly conformal dose distributions [1]. Nevertheless, the inverse planning approach that characterizes IMRT still makes compensating for possible intra-fraction (i.e., during a treatment fraction delivery) prostate motion and anatomy changes challenging. The traditional solution to account for possible uncertainties consists in irradiating a larger volume, referred as to planning target volume (PTV), generated by adding a positive margin to the original treatment volume [2]. The use of such a static margin has many limitations, which may lead to treatment ineffectiveness by overdosing the organs at risk (OAR) or underdosing the target [3,4,5,6,7,8].

**Figure 1:**
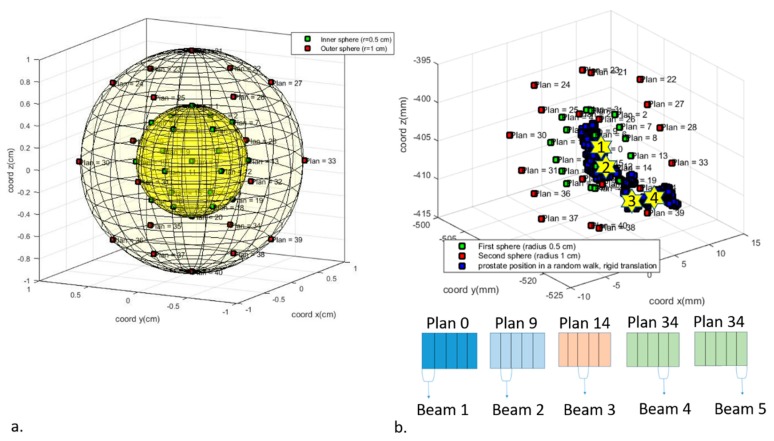
Schematic representation of the Adaptive planning method. In (**a**), the distribution of 40 possible prostate centre of mass (CM) during a treatment fraction is shown. For each CM position, a new treatment plan was generated, forming a “library of plans”. Twenty CMs are uniformly distributed at 0.5 cm and at 1 cm from the CM position in the planning CT (in green and in red, respectively). In (**b**), an example of prostate CM possible shifts within a treatment fraction is shown in blue and prostate CMs for which the “library of plans” were created are shown in green and in red. The n th yellow star indicates the prostate CM position at the end of the n th beam delivery. The minimum Euclidean distance found from that CM position and the prostate CMs of the “library of plans” determines the plan selected. The beams of the plans selected in this example are shown at the bottom of the (**b**).

While several studies developed offline/online adaptive strategies to compensate for prostate motion-induced misdosage, only a few focused on real-time intra-fraction motion management [9,10,11]. For example, Chiesa et al. used a robotic couch to rigidly shift the patient during treatment to compensate for prostate drifts [11]. However, this solution does not take into account the anatomical changes in the healthy tissue surrounding the prostate. In our recent work, we proposed instead a novel alternative solution to adapt in real time the treatment plan (i.e., a patient-specific plan including all the information relative to the treatment, e.g., dose prescription, fractionation scheme, beams to be delivered) to both possible prostate motion and tissue distribution changes of the OARs [12,13]. This strategy makes use of a pre-generated “library of plans” optimized on different tissue distributions based on a dense sampling of possible positions of the prostate during a treatment fraction (Figure 1a). The approach relies on the availability of an image guidance (IG) system to monitor the prostate location in real time [14]. The adaptive strategy consists of detecting with the IG system the current prostate position at the n th beam end (n th stars in Figure 1b) and selecting the plan in the library created for the target spatial position closest to the present one. Then the (*n* + 1) th beam of the selected plan is delivered (Figure 1b). The procedure is repeated for all the beams.

In this work, we compared conventional planning with our real-time adaptive planning method focusing on hypofractionated treatments (i.e., treatments delivered in a reduced number of treatment fractions compared with standard fractionated treatments), where misdosage occurring in individual fractions can have a high impact on the treatment efficacy [15]. We considered clinical combinations of different fractionation schemes (i.e., treatment partition over a number of treatment fractions) and different possible intra-fraction prostate shift orders of magnitude (up to 15 mm [3,4,5,16]). The aim was to test the efficacy of our approach with respect to standard IMRT in correcting for possible dose distribution errors in realistic clinical scenarios.

## 2. Results

### 2.1. Treatment Planning Methods Comparison

Table 1, Table 2, Table 3, Table 4 and Table 5 report the results for each of the fractionation schemes described in the Materials and Methods Section 4.1 and Section 4.2. The first column of each table contains the dosimetric objectives for the target (i.e., prostate and seminal vesicles) and the OARs. The second and the third columns report the corresponding dosimetric endpoints generated by the “Standard planning method” (Section 4.1.2 and Section 4.2.1) and by the “Adaptive planning method”, when prostate motion was simulated (Section 4.1 and Section 4.2), respectively. The fourth column of Table 1, Table 2, Table 3, Table 4 and Table 5, “Standard Planning Method with NO Motion”, refers to the dosimetric endpoints generated by the standard planning method created on the planning computerized tomography (CT) when no prostate motion was simulated (Section 4.1.2). For a clinically acceptable treatment delivery, the planning strategies should generate dosimetric endpoints on the target equal or higher than the prescribed dose (column 1 in Table 1, Table 2, Table 3, Table 4 and Table 5). For the OARs instead, the dose delivered should be as low as possible, or at worst equal to the referenced dose values (column 1 in Table 1, Table 2, Table 3, Table 4 and Table 5). To assess the performance of the planning methods, the dosimetric endpoints from the clinically delivered plan (Standard planning method with NO Motion) were compared with the results obtained using the “Adaptive planning method”. The “Standard planning method” was also compared with the adaptive strategy to prove that taking the prostate position into account is not sufficient to improve the dosimetric results.

Through the isodose lines, the dose distribution is projected on the patient anatomy, showing areas in which the target is not covered by the desired dose (cold spots) and regions of the OARs which instead receive a dose higher than desired (hot spots). For each fractionation scheme, the isodose lines generated by the different planning methods were examined and the presence of hot/cold spots described.

#### 2.1.1. Standard Planning Method on Simulation CT

**Figure 2:**
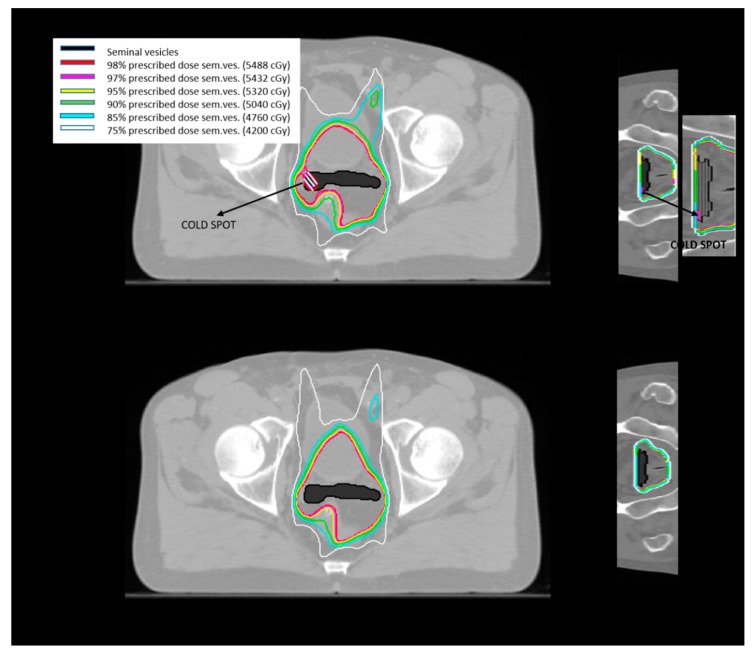
Isodose lines plot on a pelvic slice for the six-fraction scheme. (**a**) Shows the dose distribution for standard planning method, projected on the patient’s axial and coronal planes (on the left and on the right of the figure, respectively). A cold spot (white shaded area on the axial projection and highlighted on an enlarged view on the coronal projection) resulted in a region of the seminal vesicles (black area) with a dose lower than 97–98% of the dose prescription. In (**b**), the isodose lines generated by the adaptive planning method on the same pelvic slice, projected on the patient’s axial and coronal planes (on the left and on the right of the figure, respectively).

For the case of a five-fraction extreme hypofractionation with small prostate shift (see point 1.a. in the Materials and Methods Section 4.1), the treatment generated using the standard planning method was approximately equal to the planned treatment (Table 1). For a five-fraction extreme hypofractionation with larger prostate shift (point 1.b. in the Materials and Methods Section 4.1), a misdosage of about 3% of the prescribed dose was produced on the seminal vesicles (Table 2). Similar results were obtained for the schemes considering 6 and 15 fractions (points 2. and 3. in in the Materials and Methods Section 4.1), where the seminal vesicles were underdosed by 4% and 3% of the dose prescription, respectively (Table 3 and Table 4). In these fractionation schemes (points 1.b., 2., 3. in the Materials and Methods Section 4.1), cold spots below 97% of the prescribed dose were identified within the seminal vesicles contour. Figure 2a shows the isodose curves for the six-fraction scheme for the volume slice with the largest cold spot produced (shaded in white). The cold spot covered about 10% of the seminal vesicles’ volume. In the former fractionation scheme, the misdosage was generated during the fraction with a large prostate shift, underdosing the prostate and the seminal vesicles by 6% and 30% of the prescribed dose, respectively, and was only partially compensated by the following five fractions with small prostate drifts.

**Figure 3:**
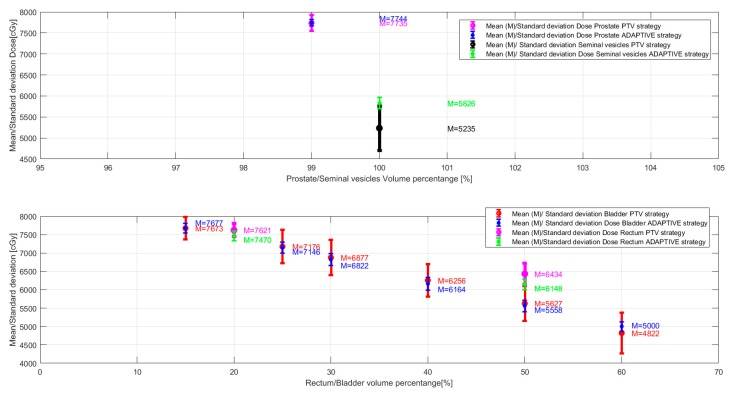
Mean/standard deviation dose values for each dosimetric endpoint (Table 1, Table 2, Table 3 and Table 4), computed over the 33 fractions simulated. Top figure: mean/standard deviation dose values for the prostate and the seminal vesicles. The values in magenta and in blue correspond to the dose absorbed by 99% of the prostate; the values in black and in green correspond to the minimum dose absorbed by the seminal vesicles. Bottom figure: mean/standard deviation dose values for the rectum and the seminal vesicles. The values in red and in blue correspond to the dose absorbed by the indicated % of the bladder volume; the values in magenta and in green correspond to the dose absorbed by the indicated % of the rectum volume.

Figure 3 shows the mean and the standard deviation of the dosimetric endpoints for the target and the OARs over the 33 fractions simulated. On average, the dose absorbed by the seminal vesicles and the rectum do not satisfy the respective dosimetric objectives. The high standard deviation present for all the dosimetric objectives reported in the figure indicates that the dose generated by this planning strategy depends on the direction and magnitude of the prostate shift during a treatment fraction.

#### 2.1.2. Adaptive Planning Method on Simulation CT

For the adaptive planning method, in all fractionation schemes taken into account, the dosimetric objectives for both the target and the OARs were always satisfied (Table 1, Table 2, Table 3 and Table 4). Comparing the different fractionation schemes, the dosimetric distributions generated were very similar, with dose variations always smaller than 1 Gy: the mean variation per objective between fractionation schemes ranged from 18 cGy to about 63 cGy. The same trend was observed in the individual fractions simulations. Figure 3 shows that the dosimetric endpoints obtained for fractions where different prostate shifts were simulated are on average always clinically acceptable (as they satisfy the dosimetric objectives) and have a small standard deviation. This means that regardless of the magnitude and the direction of the prostate shift during a treatment fraction, the adaptive planning method is robust and can deliver a clinically acceptable dose to the target and the OARs.

For example, for the six-fraction scheme, the dose resulting from the treatment fraction with prostate large displacement was similar to those fractions in which prostate displacement was small. Figure 2b shows the isodose curves produced on the seminal vesicles on the volume slice where the largest cold spot was generated applying the standard planning approach (Figure 2a). The dose resulted to be uniformly distributed on the organ, with no cold spots present.

Compared with the traditional planning method, the dose on the rectum was lowered on average by 3–4% for each dosimetric endpoint in all the fractionation schemes considered (Table 1, Table 2, Table 3 and Table 4).

#### 2.1.3. Standard Planning Method VS. Adaptive Planning Method on CBCT

Table 5 compares the dosimetric endpoints obtained using the Standard planning method and the Adaptive planning method on the treatment fraction described in Section 4.2, where the patient cone beam computerized tomography (CBCT) has been used to simulate intra-fraction tissue changes. In this case, the standard planning method delivered a correct dose to the target and OARs. The adaptive planning method reduced drastically the dose on the OARs; while delivering a clinically acceptable dose to the target.

## 3. Discussion

Different studies tried to quantify the impact of intra-fraction prostate motion on treatment outcome when a treatment margin is used [7,17,18]. In general, as more treatment fractions are considered, the dose errors due to prostate motion become less relevant. In fact, when the dose is partitioned over a significant number of fractions, the dose delivered in every single fraction is relatively small (e.g., 2 Gy) compared with the total treatment dose (e.g., 40 Gy). The random nature of prostate motion in combination with a significant number of fractions may result in compensation of dosimetric errors between different fractions. However, in hypofractionated schemes, prostate motion can have a much larger impact on the treatment efficacy due to the increased dose delivered in each fraction, possibly underdosing the target and overdosing the rectum [19].

In our work, we analysed how the dose distribution is affected by prostate motion in different fractionation schemes (including extreme hypofractionation), comparing results for the traditional planning method and the adaptive planning approach developed. The combinations of prostate shifts we used in this work were clinically reasonable. They were based on studies in literature where volumetric real-time tracking of the target was performed with ultrasound guidance systems [3,16]. The largest shift used (13 mm) was reported in those studies and it was simulated in our work on one single occurrence over 33 treatment fractions [3,16].

For the traditional approach, a lack of control on dose deposition was observed, due to the PTV margin overlapping with different portions of the surrounding OARs depending on the specific motion of the prostate. Frequent misdosage was observed on the seminal vesicles, suggesting that this planning method is suboptimal when significant intra-fraction changes occur in hypofractionated treatments. Cold spots in the seminal vesicles were observed in almost every case analysed, indicating poor dose coverage in a subregion of the organ. The effect of suboptimal dose delivery on tumour control probability (TCP) can be significant even for small cold spots, as showed by [20]; in particular, for a situation like the one introduced in our work, that is, a 3% or 4% underdosage of the whole target, they predict up to almost 15% decrease in TCP, in some scenarios.

The adaptive approach tested in this work, instead, does not rely on the randomness of prostate movement over many fractions to compensate for possible errors, but it adapts the beam to the tissues distribution in real time. The dose distributions generated showed very small variations among the treatment fractions also when the prostate shifts were significantly different (e.g., 1 mm shift versus 10 mm), indicating that the dose absorption was almost independent of prostate movements. This was also confirmed by the negligible variation in dose distribution for the different fractionation schemes. The additional tests performed using a patient CBCT to simulate intra-fraction tissue changes confirmed the ability of this planning method to adapt to the tissue distribution during treatment delivery and to deliver a reduced dose to the OARs.

The adaptive planning strategy ability to adapt to intra-fraction changes has important implications not only for the target but also for the rectum. Several studies proved the relationship between the rate of acute rectum toxicity and the rectum volume irradiated [19,21,22], showing that the dose relative to the rectum dosimetric endpoints was significantly higher for patients experiencing toxicity. The combination of adaptive approach here introduced and image guided radiation therapy (IGRT)/IMRT can be used to further reduce the dose deposited on the rectum and thereby reduce acute rectum toxicity.

## 4. Materials and Methods

### 4.1. Treatment Fractions on Simulation CT

A traditional IMRT planning method and the proposed adaptive planning strategy were applied on the clinical simulation CT of a patient with intermediate-risk prostate cancer, locally extending to the seminal vesicles. Thirty-three possible prostate shifts (ranging from 1 mm to 15 mm, based on the findings of Balhausen et al. [3,16]) occurring during treatment fractions were simulated, deforming elastically the CT scan, as described in Section 4.1.2.

The following three fractionation schemes were simulated using the 33 simulated shifts, and corresponding dosimetric endpoints were calculated taking prostate motion into account:

1. IMRT extreme hypofractionation five-fraction scheme for a total dose of 80 Gy (16 Gy per fraction), where the five fractions were selected from the 33 available according to the following two different criteria:
  - Five fractions randomly selected among the ones with small prostate motion (0–3 mm) in the set of the 33 simulated;
  - Five fractions randomly selected among the ones with intermediate magnitude prostate motion (3.5–7.5 mm) in the set of the 33 simulated.
2. IMRT extreme hypofractionation six-fraction scheme for a total dose of 80 Gy (~13 Gy per fraction), combining one treatment fraction with a large prostate shift (13 mm) from the 33 available with the five-fraction scheme in 1a;
3. IMRT hypofractionation 15-fraction scheme for a total dose of 80 Gy (~5.33 Gy per fraction), adding to the six-fraction scheme in the previous point (2.). Eight fractions with a prostate displacement of intermediate magnitude (3–8 mm), randomly selected among the 33 available.

The key steps implemented for the treatment strategy simulations are extensively explained in [12]. A summary is reported in the next Sections (Section 4.1) [12].

#### 4.1.1. Treatment Simulation for the Standard Planning Strategy

An IMRT reference plan with 5 mm PTV margin was created using a research version of the Philips Pinnacle^3^ treatment planning system (TPS) (version 16.0 Philips Radiation Oncology Systems, Fitchburg, WI, USA) [23]. The plan was optimized using five non-pairwise opposed photon beams (0, 30, 135, 225, 330 deg) and standard dosimetric values (see Table 5), delivering a total dose of 80 Gy [24,25].

**Figure 4:**
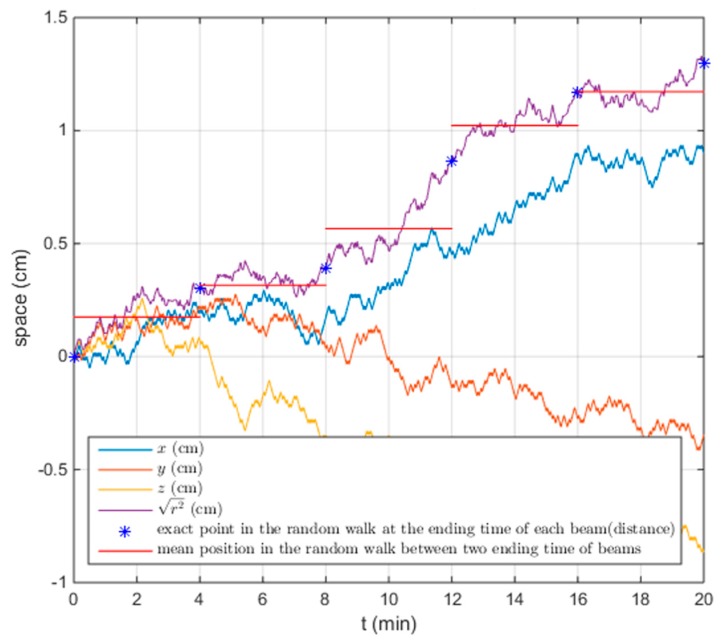
Matlab plot of an example of prostate CM Random walk in a time interval of a 20 min, where each beam is delivered for 4 min. Six hundred prostate positions were sampled (30 steps per minute), adding a fixed distance in a random direction to the previous CM position ([3,16]). The blue stars in the figure represent the exact positions of the CM at the ending time of each beam delivery, and the red line is the mean CM position during the delivery time of the corresponding beam.

A three-dimensional (3D) random walk model was implemented in MatLab (version 2014, The MathWorks, Inc., Natick, MA, USA) in order to simulate the prostate motion during each treatment fraction [3,16]. The prostate center of mass (CM) at the treatment beginning was assumed to coincide with the reference CM position on the planning CT. The next CM position in time was computed by adding a fixed distance in a random direction to the previous CM position, and so on. Considering a 20 min treatment, 600 prostate positions were sampled for each fraction simulated (Figure 4). Figure 4 shows an example of simulated Random walk, with the prostate CM travelling distance for each beam in a treatment fraction the average CM position was then computed, assuming that within a treatment fraction each beam would be delivered for 4 min (red lines in Figure 4). The planning CT was then modified to represent the tissue configuration during each beam delivery according to the random path computed. The planning CT was then modified to represent the tissue configuration during each beam delivery according to the random path computed. Thus, for each beam in a treatment fraction, a new simulation CT was generated. This was performed by rigidly shifting the prostate to the average CM position computed for the respective beam and elastically deforming the OARs accordingly. The procedure to create the simulation CTs is detailed in the following steps:

- The prostate in the planning CT was surrounded by a minimum encompassing radius that was subsequently expanded isotropically by 2 cm. This surface enclosed the image region in which the original tissue distribution had to be adjusted to represent the new tissue configuration; while all the voxels outside this region remained as in the original image. For this reason, we refer to this surface as “zero motion barrier” (ZMB).
- A motion vector field (MVF) was determined to map the original voxel centres positions to the new configuration. The voxels of the prostate were rigidly translated according to the new position of the prostate CM. A 3D thin-plate spline interpolation was then computed between the remaining pixels in the ZMB and the pixels surrounding the ZMB.
- To generate the modified planning CT, a new grid was generated having the same dimensions as the original image. An inverse motion vector field (IMVF) was computed in MatLab to determine the relation between the new grid and the original image. The IMVF was applied to the voxels of the new grid. Whenever a voxel in the new grid corresponded to the voxel center in the original image, its voxel intensity was set as the intensity of the respective voxel in the original image. For the voxels in the new grid corresponding to “off-grid” points, their intensity values were computed performing a trilinear interpolation of the intensity values corresponding to the neighbouring voxels in the original image.

The approximate dose delivered per beam was computed using the Adaptive Convolve Engine [26] in the TPS with a dose grid resolution of 0.4 cm on the modified CT scan. The dose distributions generated by each beam were summed for each treatment fraction. The dose distributions obtained for all the fractions composing a fractionation scheme were then added and rescaled to the number of fractions in the fractionation scheme. The respective dose–volume histogram (DVHs) was then generated.

#### 4.1.2. Treatment Simulation for the Adaptive Planning Strategy

Forty equidistant points representing possible positions of the prostate were sampled on two spheres, one at 5 mm and the other at 10 mm from the prostate centre of mass (CM) in the planning CT [27]. For each of the sampled points, a new CT volume was generated by modifying the original planning CT by rigidly shifting the prostate to the new position and deforming the surrounding organs accordingly. The new CTs were generated following the same steps reported in Section 4.1.2. A library of plans was created in the TPS, optimizing a new plan for each of the modified CT volumes, with the same plan characteristics and dosimetric objectives as in Section 4.1.2 and the clinical target volume (CTV) as treatment target (thus with no treatment margin applied). For the original CT, the IMRT reference plan was re-optimized, this time on the CTV.

The prostate shifts during treatment were then simulated using the random walk model, as described in Section 4.1.2. While with the conventional approach the plan prepared for the planning CT is delivered regardless to the prostate positions during treatment, with the adaptive approach each of the beams is selected based on the current location of the prostate. Assuming the patient is aligned with the planning CT, the initial step was to deliver the first beam of the plan created for the original CT. At the end of the first beam delivery, a minimization algorithm found the sampled point with the smallest Euclidean distance to the current prostate CM. The second beam of the corresponding plan in the library was then selected and delivered. This process was repeated at each beam end until treatment fraction end. As in Section 4.1.2, the dose delivered by each of the beams was computed in the TPS, delivering the beam selected on the simulation CT, representing the prostate and OARs configuration during the specific beam.

As introduced in Section 4.1.2, the dose distributions generated by each beam were summed for each treatment fraction. For each fractionation scheme, the doses obtained for each treatment fraction were first added together and then rescaled to the number of fractions in the fractionation scheme. Finally, the DVH for each fractionation scheme was computed.

### 4.2. Treatment Fractions on CBCT

The standard planning method and the adaptive planning method were compared on an additional prostate cancer patient with intermediate-risk prostate cancer CT. A CBCT of the patient was obtained prior to a treatment fraction delivery for re-planning. The prostate shift on the CBCT was about 0.5 mm. In this work, the CBCT was used to represent the tissue distribution due to a possible prostate drift occurring during a treatment fraction. During the delivery of the first beam, the prostate CM was assumed to be aligned with the planning CT; while for the subsequent beams, the tissue distribution was represented by the CBCT, and thus the prostate CM coincided with the one on the CBCT.

#### 4.2.1. Treatment Simulation for the Standard Planning Strategy

An IMRT plan with the same characteristics and planning objectives as in Section 4.1.2 was created for the patient CT. A new CT was generated from the CBCT for dose computation purposes. The CBCT voxel dimensions were rescaled to the patient CT. In order to match the CT to the CBCT tissue distribution, the CT was then deformed elastically on the CBCT by applying a B-spline deformation in 3D Slicer. The ROIs used for planning on the CT were then propagated onto the corresponding organs on the new CT generated. This procedure was implemented in the TPS, and the contours produced approved by an experienced clinician. The dose distribution during the treatment fraction was computed in the TPS as described in Section 4.1.2, utilizing the CT for the first beam and the new CT based on the CBCT for the consecutive beams.

#### 4.2.2. Treatment Simulation for the Adaptive Planning Strategy

The library of plans for this additional patient was created as described in Section 4.1.2. For the patient CT, the IMRT standard plan was re-optimized on the CTV as in Section 4.1.2. The dose distribution delivered by the first beam of this plan was computed on the planning CT, since it was assumed that during the first beam delivery the patient would be aligned with the planning CT. For the consecutive beams, the distance between the prostate CMs on the new CT representing the CBCT tissue configuration (see Section 4.2.1) and the CMs in the library of plans were computed, and the plan corresponding to the closest CM in space was selected. The dose distributions were then computed in the TPS using the beams of the selected plan and the new CT.

## 5. Conclusions

Traditional IMRT planning approaches rely on statistical compensation of possible misdosage errors over several fractions. For hypofractionated treatments, the compensation is less likely to occur, especially in case large prostate shifts occur. In our study, we showed that the traditional planning strategy can result in underdosing the seminal vesicles and overdosing the rectum. The proposed real-time correction, instead, generated a dose distribution almost insensitive to intra-fraction prostate drifts or fractionation schemes that was in agreement with the dosimetric requirements in every case analysed. Compared with the traditional planning strategy, the adaptive planning strategy reduced significantly the dose on the rectum.

## 6. Patents

This work is related to an invention previously patented by two of the co-authors: Davide Fontanarosa; Alfonso Agatino Isola, patent #WO 2017005758 A1, 12/01/2017.

## Figures and Tables

**Table 1:** IG-IMRT (image guided intensity-modulated radiation therapy) extreme hypofractionation treatment five fractionation scheme: prostate small motion. Target and OAR (organs at risk) doses for the volume fractions for which the requirements reported in the Materials and Methods (Table 6) were imposed, for the standard IMRT treatment.

	Dose [cGy]		
	Standard Planning Method	Adaptive Planning Method	Standard Planning Method No Motion
Prostate D99% > 7600 cGy	7957	7809	7952
Seminal vesicles D min > 5600 cGy	5508	5777	5594
Bladder V5000 cGy < 60%	5154	5098	5184
Bladder V6500 cGy < 50%	5925	5685	5942
Bladder V6600 cGy < 40%	6566	6247	6595
Bladder V7000 cGy < 30%	7169	6910	7205
Bladder V7500 cGy < 25%	7508	7248	7580
Bladder V8000 cGy < 15%	7877	7766	7908
Rectum V6170 cGy < 50%	6383	6049	6382
Rectum V7429 cGy < 20%	7602	7397	7632

Note: the dose values for the objective on the prostate (D3) and those for the femurs are not reported as the dosimetric objectives were always satisfied in our analysis.

**Table 2:** IG-IMRT extreme hypofractionation treatment, five fractionation scheme: prostate intermediate increasing motion. Target and OAR doses for the volume fractions for which the requirements reported in the Materials and Methods (Table 6) were imposed for the standard IMRT treatment.

	Dose [cGy]		
	Standard Planning Method	Adaptive Method	Standard Planning Method No Motion
Prostate D99% > 7600 cGy	7940	7838	7952
Seminal vesicles D100% > 5600 cGy	5410	5851	5594
Bladder V5000 cGy < 60%	4865	5003	5184
Bladder V6500 cGy < 50%	5717	5587	5942
Bladder V6600 cGy < 40%	6265	6174	6595
Bladder V7000 cGy < 30%	6970	6854	7205
Bladder V7500 cGy < 25%	7294	7200	7580
Bladder V8000 cGy < 15%	7757	7736	7908
Rectum V6170 cGy < 50%	6448	6111	6382
Rectum V7429 cGy < 20%	7631	7465	7632

Note: the dose values for the objective on the prostate (D3) and those for the femurs are not reported as the dosimetric objectives were always satisfied in our analysis.

**Table 3:** IG-IMRT extreme hypofractionation treatment, six-fraction scheme. Target and OAR doses for the volume fractions for which the requirements reported in the Materials and Methods (Table 6) were imposed, for the standard IMRT treatment.

	Dose [cGy]		
	Standard Planning Method	Adaptive Method	Standard Planning Method No Motion
Prostate D99% > 7600 cGy	7887	7795	7952
Seminal vesicles D100% > 5600 cGy	5337	5804	5594
Bladder V5000 cGy < 60%	4908	5085	5184
Bladder V6500 cGy < 50%	5772	5658	5942
Bladder V6600 cGy < 40%	6298	6231	6595
Bladder V7000 cGy < 30%	6973	6879	7205
Bladder V7500 cGy < 25%	7256	7220	7580
Bladder V8000 cGy < 15%	7712	7737	7908
Rectum V6170 cGy < 50%	6413	6056	6382
Rectum V7429 cGy < 20%	7621	7386	7632

Note: the dose values for the objective on the prostate (D3) and those for the femurs are not reported as the dosimetric objectives were always satisfied in our analysis.

**Table 4:** IG-IMRT hypofractionation treatment 15 fractionation scheme. Target and OAR doses for the volume fractions for which the requirements reported in the Materials and Methods (Table 6) were imposed, for the standard IMRT treatment.

	Dose [cGy]		
	Standard Planning Method	Adaptive Method	Standard Planning Method No Motion
Prostate D99% > 7600 cGy	7823	7823	7952
Seminal vesicles D100% > 5600 cGy	5419	5812	5594
Bladder V5000 cGy < 60%	4938	5032	5184
Bladder V6500 cGy < 50%	5783	5607	5942
Bladder V6600 cGy < 40%	6356	6171	6595
Bladder V7000 cGy < 30%	6999	6849	7205
Bladder V7500 cGy < 25%	7321	7183	7580
Bladder V8000 cGy < 15%	7750	7717	7908
Rectum V6170 cGy < 50%	6423	6118	6382
Rectum V7429 cGy < 20%	7625	7442	7632

Note: the dose values for the objective on the prostate (D**3**) and those for the femurs are not reported as the dosimetric objectives were always satisfied in our analysis.

**Table 5:** IG-IMRT treatment fraction on CBCT. Target and OAR doses for the volume fractions for which the requirements reported in the Materials and Methods (Table 6) were imposed for the standard IMRT treatment.

	Dose [cGy]		
S	Standard Planning Method	Adaptive Method	Standard Planning Method No Motion
Prostate D99% > 7600 cGy	7748	7666	7943
Bladder V5000 cGy < 60%	4970	904	5219
Bladder V6500 cGy < 50%	5784	1685	6068
Bladder V6600 cGy < 40%	6595	3028	6754
Bladder V7000 cGy < 30%	7288	4759	7393
Bladder V7500 cGy < 25%	7598	5549	7658
Bladder V8000 cGy < 15%	7888	7030	7954
Rectum V6170 cGy < 50%	5779	4984	6245
Rectum V7429 cGy < 20%	7334	7174	7441

Note: the dose values for the objective on the prostate (D**3**) and those for the femurs are not reported as the dosimetric objectives were always satisfied in our analysis.

**Table 6:** Dosimetric objectives on treatment volume and OARs for the whole treatment. The following conventions were used: Dv% > [d] Gy means that the dose that covered *v%* of the volume must be bigger than *d*Gy; V[d] Gy < [v]% means that the volume fraction that received at most *d*Gy must be smaller than *v%* of the volume.

		DOSIMETRIC OBJECTIVES
TREATMENT VOLUME	PTV\CTV a	D99% > 7600 cGy
	PTV\CTV a	D3% < 8300 cGy
	PTV\CTV a	D uniform = 8000 cGy
	SEMINAL VESICLES	D min > 5600 cGy
OAR	BLADDER	V5000 cGy < 60%
	BLADDER	V6500 cGy < 50%
	BLADDER	V6600 cGy < 40%
	BLADDER	V7000 cGy < 30%
	BLADDER	V7500 cGy < 25%
	BLADDER	V8000 cGy < 15%
	RECTUM	V6170 cGy < 50%
	RECTUM	V7429 cGy < 20%
	LEFT FEMUR	D max < 4000 cGy
	RIGHT FEMUR	D max < 4000 cGy

Note: The dosimetric objective refers either to the PTV (planning target volume) or to the clinical target volume (CTV), depending on the study case.

## Abbreviations

EBRT	External Beam Radiation Therapy
IMRT	Intensity-Modulated Radiation Therapy
PTV	Planning Target Volume
OAR	Organ at Risk
IG	Image Guidance
CT	Computerized Tomography
TPS	Treatment Planning System
CTV	Clinical Target Volume
DVH	Dose-Volume Histogram
CM	Centre of Mass
